# Genistein Induces Ferroptosis in Colorectal Cancer Cells via FoxO3/SLC7A11/GPX4 Signaling Pathway

**DOI:** 10.7150/jca.95775

**Published:** 2024-11-04

**Authors:** Longfei Liu, Yuan Qiu, Zehao Peng, Zhongchao Yu, Hengzhe Lu, Rongjun Xie, Zhongcheng Mo, Sen Zhang

**Affiliations:** 1 Department of Colorectal & Anal Surgery, The First Affiliated Hospital of Guangxi Medical University, Nanning 530021, China.; 2 The Affiliated Nanhua Hospital, Department of Gastrointestinal Surgery, Hengyang Medical College, University of South China, Hengyang, Hunan 421001, China.; 3 Guangxi Key Laboratory of Diabetic Systems Medicine, Department of Histology and Embryology, Guilin Medical University, Guilin 541199, Guangxi, China.

**Keywords:** Genistein, Colorectal cancer, Ferroptosis, FoxO3.

## Abstract

Colorectal cancer is among the most frequently diagnosed cancers with high mortality rates and poses a serious threat to human health. Genistein (Gen) has been found to have anti-colorectal cancer effects, however, the molecular mechanisms by which genistein elicits its effects on colorectal cancer (CRC) cells have not been fully elucidated. In this study, we investigated the oxidative state of colorectal cancer cells during the antitumor action of Genistein and whether it can exert its antitumor effects through ferroptosis. Current research on the oxidative state of Genistein indicates that it exhibits both antioxidant and pro-oxidant properties. Different drug concentrations were applied to colorectal cancer cells, after which cell viability and key markers of ferroptosis, including reactive oxygen species (ROS), malondialdehyde (MDA), and Fe^2+^, were measured. We found that genistein significantly reduced the viability of colorectal cancer cells, and the expression of ferroptosis markers increased in a concentration-dependent manner. Subsequently, we treated cells with the ferroptosis inhibitor fer-1 in combination with genistein and observed a partial reversal of ferroptosis markers. These findings suggest that genistein exerts its antitumor effect by promoting iron-dependent oxidative damage-induced ferroptosis. To further elucidate the mechanism underlying ferroptosis modulation, we examined the protein and mRNA expression levels of the classical key ferroptosis molecules SLC7A11 and GPX4. We found that the expression levels of these molecules decreased, with GPX4 exhibiting a greater decrease. Overexpression of GPX4 reversed the pro-ferroptotic effect of genistein, indicating that genistein promotes ferroptosis occurrence by downregulating GPX4 expression. When the drug was applied to colorectal cancer cells, the expression of the transcription factor FoxO3 increased. Treatment of cells with the FoxO3 inhibitor JY-2 in combination with other drugs resulted in antagonism of ferroptosis markers. These findings suggest that genistein induces ferroptosis in colorectal cancer cells through the FoxO3/SLC7A11/GPX4 signaling pathway, thereby inhibiting tumor growth.

## 1. Introduction

Colorectal cancer (CRC) is one of the most common and deadly cancers worldwide, and its incidence is increasing annually. With the popularity of screening methods, an increasing number of young people are being diagnosed with CRC 1. In the United States, the incidence of CRC in adults aged >20-49 years has increased by nearly 45% 2. The reasons for this increasing incidence are associated with unhealthy lifestyles, dietary habits, and underlying diseases, including smoking, alcohol consumption, sedentary behavior, a Western diet, and diabetes 3-6. Shockingly, individuals under 50 years old with diabetes have a 27% greater risk of developing tumors than healthy individuals 7. Moreover, due to late-stage diagnosis in the majority of CRC patients 8, the optimal surgical window is missed, and the efficacy of existing chemotherapy regimens is limited for metastatic CRC 9. Therefore, there is a need to explore new chemotherapeutic methods to achieve better outcomes and reduce the incidence and mortality rate of CRC. Dietary supplements have attracted attention because they can enhance physical fitness and reduce the probability of developing disease 10. Dietary supplements are substances that contain nutritional or physiologically active ingredients, including plant or herbal extracts, vitamins, minerals, amino acids, etc. These supplements also have antioxidant, anti-inflammatory, anticancer, and immune regulatory functions and play important roles in cancer prevention and prognosis. Studies have shown that dietary supplements can prevent carcinogenesis and inhibit the growth of CRC cells 11, thus potentially providing new strategies for CRC treatment.

Genistein (Gen) is a natural isoflavone that can be extracted from various plants 12. It not only has anticancer activity but also has multiple biological effects, such as antidiabetic, anti-inflammatory, and antioxidant effects. Among these, its anticancer activity has been extensively studied in breast cancer, CRC, gastric cancer, and liver cancer, among others 13. Rasheed, S's study indicated that consuming Gen in the diet can reduce the risk of CRC by 23% 14. Therefore, Gen holds great research value in the prevention and treatment of CRC. Research has suggested that Gen achieves its effects on CRC prevention and treatment by inhibiting cell proliferation, invasion, and migration, blocking the progression of the cell cycle at the G2/M phase 15, inhibiting angiogenesis 16, exerting antioxidant effects 17, suppressing inflammation 18, promoting apoptosis 19, inhibiting topoisomerase II 20, and activating estrogen receptors 21. Several studies suggest that Gen has antioxidant effects, stabilizes cellular antioxidant status, and scavenges oxygen free radicals, thereby inhibiting carcinogenesis by preventing oxidative damage in the tissue microenvironment 17, 22. However, research has also shown that Gen can weaken tumor proliferation by promoting oxidative damage in prostate cancer 23 and acute myeloid leukemia in humans 24. The latest research on Gen in CRC indicates that treatment with Gen leads to a significant increase in endogenous reactive oxygen species (ROS) in SW620 cells, and the accumulation of ROS leads to cell death, which is caused by decreased expression of antioxidant enzymes and weakened capacity to scavenge oxygen free radicals 25. Therefore, our study aimed to further explore the dynamic process of oxidative stress during the anticancer effects of Gen in CRC and whether this process tends toward oxidative accumulation or an antioxidative state.

Ferroptosis is a programmatic cell death process driven by iron-dependent lipid peroxidation accumulation 26. It plays a crucial role in the treatment of various cancers, not only by directly inducing tumor cell death but also by reversing the drug resistance of tumors to chemotherapy 27-29, thus enhancing the therapeutic efficacy of chemotherapy drugs. Therefore, ferroptosis is considered an effective target for treating different types of cancer. However, the mechanism of action of genistein, a compound that has anticancer properties, is complex, and it is unclear whether it triggers ferroptosis. Interestingly, ferroptosis is characterized by lipid peroxidation accumulation and an impact on antioxidant enzymes, which aligns with the changes observed in cells upon the introduction of genistein. Therefore, it is reasonable to speculate that genistein could exert its anticancer effects through ferroptosis. This study sought to investigate whether genistein can exert anticancer effects through ferroptosis and how it influences ferroptosis in CRC cells.

## 2. Material and Methods

### 2.1 Cell culture and treatment

Human colorectal cancer cell lines HCT116 and HT-29 were obtained from the Chinese Academy of Sciences (Shanghai, China). The cells were cultured in DMEM (#PYG0073, Boster, China) and McCoy's 5A (#PYG0025, Bode Biology, China) media supplemented with 10% fetal bovine serum (#11011-8611, Zhejiang Tianhang, China), 100 IU/mL penicillin, and 100 IU/mL streptomycin in a humidified incubator at 37°C with 5% CO_2_. Genistein (#G0272; Tokyo Chemical Industry Co., Ltd.), the ferroptosis inhibitor ferrostatin-1 (#HY-100579; MedChemExpress), and JY-2 (#HY-153347; MedChemExpress) were dissolved in dimethyl sulfoxide (DMSO). At specified concentrations and time points, the cells that reached 70% confluency were treated with these compounds, and the DMSO concentration was maintained below one thousandth throughout the treatment process.

### 2.2 Cell viability

Cell viability was assessed using a Cell Counting Kit-8 (#AR1160, Boster, China). Cancer cells were grown in 96-well plates at a density of 5000 cells/well. After treatment, 10 μL of CCK-8 solution was added, and the mixture was incubated at 37 °C for 1 h. The absorbance was measured at 450 nm using a microplate reader, and each experiment was repeated three times.

### 2.3 Levels of Fe^2+^ and MDA

The MDA and Fe^2+^ contents of the treated cells were analyzed using a Lipid Peroxidation MDA Assay Kit (#S0131S; Shanghai Biyun Tian, China) and a Ferrozine Colorimetric Assay Kit for Iron Detection (#ADS-W-QT027; Jiangsu Feiya, China), respectively, according to the manufacturer's instructions. All the results were normalized to the corresponding total cell numbers.

### 2.4 ROS levels

Intracellular ROS levels in HCT116 and HT-29 cells treated with the indicated compounds were determined using the oxidation-sensitive fluorescent probe dichlorodihydrofluorescein diacetate (DCFH-DA) (#E004-1-1; Nanjing built, China). A fluorescence microscope (Bio-Rad, Singapore) was used for photographic processing.

### 2.5 Western blotting

The logarithmic growth phase cells were seeded in a six-well plate at a density of 500,000 cells per well. After treatment, the cells were collected and lysed in RIPA (#R0020, Solarbio China) buffer. The total protein concentration of each sample was determined using a BCA protein concentration assay kit (#AR0146, Boster, China). Equal amounts of protein from each sample were separated via 10% sodium dodecyl sulfate‒polyacrylamide gel electrophoresis and transferred to a PVDF membrane. Next, the membrane was blocked with 5% nonfat milk at room temperature for 1 h and then incubated with primary antibodies against GPX4 (#BM5231), SLC7A11 (#BM5318), FoxO3 (#PB9196), and β-actin (#BM0627) overnight at 4 °C. All antibodies were purchased from Boster (Wuhan, China) and diluted 1:1000. The membranes were then incubated with HRP-conjugated anti-mouse IgG (#BA1050, 1/10000) and HRP-conjugated anti-rabbit IgG (#BA1054, 1/10000) secondary antibodies at room temperature for 1 h, followed by band visualization using a Universal Hood Ⅱ (Bio-Rad, USA) and analysis of the results with ImageJ software.

### 2.6 RT‒qPCR

Total RNA was extracted using TRNzol (#DP424, TIANGEN, Beijing, China), and cDNA was synthesized by reverse transcription using the FastKing One Step RT‒qPCR Kit (#KR118, TIANGEN, Beijing, China). Real-time quantitative PCR (RT‒qPCR) was performed using RealUniversal Color Fluorescent Quantitative SYBR Green Premix (Cat#FP201; TIANGEN, Beijing, China) on a CFX Connect Real-Time System (Bio-Rad, Singapore) data analysis system. The PCR procedure was as follows: 1 cycle of 95 °C for 15 min, followed by 40 cycles of 95 °C for 10 s and 60 °C for 30 s. GAPDH was used as an internal reference gene. The relative expression levels of the transcripts were calculated using the 2-ΔΔCT method. The sequences of primers used were as follows: GPX4 (forward: 5′-AGAGATCAAAGAGTTCGCCGC-3′, reverse: 5′-TCTTCATCCACTTCCACAGCG-3′), SLC7A11 (forward: 5′-GCTGTGATATCCCTGGCATT-3′, reverse: 5′-GGCGTCTTTAAAGTTCTGCG-3′), FoxO3 (forward: 5'- ATGGCAGAGGCACCAGCCTCC-3', reverse: 5'- TCAGCCTGGTACCCAGCTTTG-3′), and GAPDH (forward: 5′-CCAGGTGGTCTCCTCTGA-3′, reverse: 5′-GCTGTAGCCAAATCGTTGT-3′). All the reagents were purchased from TIANGEN (Beijing, China).

### 2.7 Plasmid transfection

The cloning vector PcDNA3.1(+) with the cloning sites KPNI and EcoRI, designated Y5844, was obtained from the host bacterium Top10. Both the plasmid and the control vector were purchased from GenePharma (Suzhou, China). For cell transfection, Lip3000 reagent (#BL632B; Biosharp, China) was used. After transfection, the transfection medium was replaced with regular culture medium containing Gen after 6h. Total protein and total RNA were extracted 72h later.

### 2.8 Hematoxylin-eosin staining and immunohistochemistry

Hematoxylin and eosin (HE) staining and immunohistochemistry (IHC) were performed using standard methods. The primary antibody used was against GPX4 (1:100 dilution; #BM5231; Abcam), which was incubated overnight at 4 °C. Staining was performed using a Universal Two-Step Test Kit (Mouse/Rabbit) (#PV-9000, Zhongshan Golden Bridge, China) and a DAB Staining Kit (#ZLI-9018, Zhongshan Golden Bridge, China). Images were randomly taken from 3 samples in each group and captured using an inverted microscope for analysis.

### 2.9 Statistical analysis

The Western blots were processed for grayscale analysis using ImageJ2x. The statistical significance of differences was analyzed using one-way ANOVA. Statistical analysis was performed using GraphPad Prism (version 8.0.2, GraphPad Software). All the data are presented as the means ± standard deviations from three independent experiments. p < 0.05 was considered to indicate statistical significance.

## 3. Results

### 3.1 Gen exerts its antitumor effect by inducing ferroptosis in CRC cells

**Figure 1:**
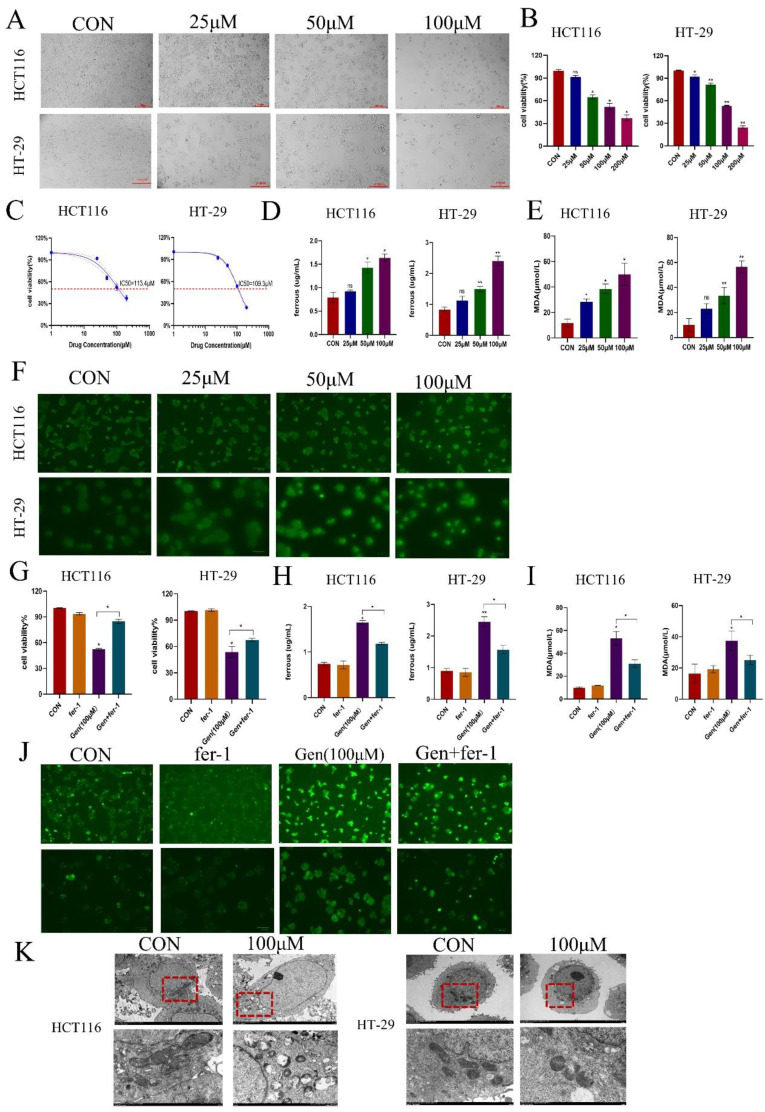
** Gen exerts antitumor effects by promoting ferroptosis in CRC cells. (A)** Results of inverted microscope shots taken 72 h after treatment of cells with Gen. **(B)** A CCK-8 assay was used to determine the viability of HCT116 and HT-29 cells after 72 h of Gen treatment. **(C)** The IC50 of Gen on HCT116 and HT-29 cells was determined via the CCK-8 assay. (D-F) Relative levels of Fe^2+^**(D)**, ROS **(E)**, and MDA **(F)** in HCT116 and HT-29 cells after 72 h of treatment with different Gen concentrations. **(G)** Cell viability was determined by a CCK-8 assay in which cancer cells were pretreated with the ferroptosis inhibitor fer-1 (10 μM) for 12 h and subsequently treated with Gen (100 μM) for 72 h. **(H-J)** Relative levels of intracellular Fe^2+^ **(H)**, MDA **(I)**, and ROS **(J)** in cancer cells pretreated with the ferroptosis inhibitor fer-1 (10 μM) for 12 h and subsequently treated with Gen (100 μM) for 72 h. **(K)** Morphological changes in mitochondria in the control and 100 μM drug groups were observed via transmission electron microscopy. The data are presented as the means ± SDs (n=3); *p<0.05; **p<0.01.

To investigate the effect of Gen on cell proliferation, we treated HCT116 and HT-29 cells with different concentrations of Gen for 72 h. After treatment with Gen at concentrations ranging from 25 to 200 μM, the viability of the HCT116 and HT-29 cells significantly decreased in a concentration-dependent manner (Figure 1A-B). The IC50 values for HCT116 and HT-29 cells were 113.4 μM and 108.1 μM, respectively (Figure 1C). Experiments conducted with 25, 50, and 100 μM Gen demonstrated that this natural compound upregulated the levels of Fe^2+^, MDA, and ROS in the cells (Figure 1D-F). Additionally, the addition of the ferroptosis inhibitor fer-1 in the presence of the drug significantly restored cell viability (Figure 1G) and led to a decrease in Fe^2+^, MDA, and ROS levels to varying degrees (Figure 1H-J). Furthermore, via transmission electron microscopy, it was evident that cells treated with 100 μM Gen exhibited noticeable mitochondrial damage, including mitochondrial ridge shrinkage, swelling, and even rupture (Figure 1K). Therefore, Gen appears to inhibit the growth of colorectal cancer cells by inducing ferroptosis.

### 3.2 Gen promotes ferroptosis in CRC cells by affecting SLC7A11/GPX4

**Figure 2:**
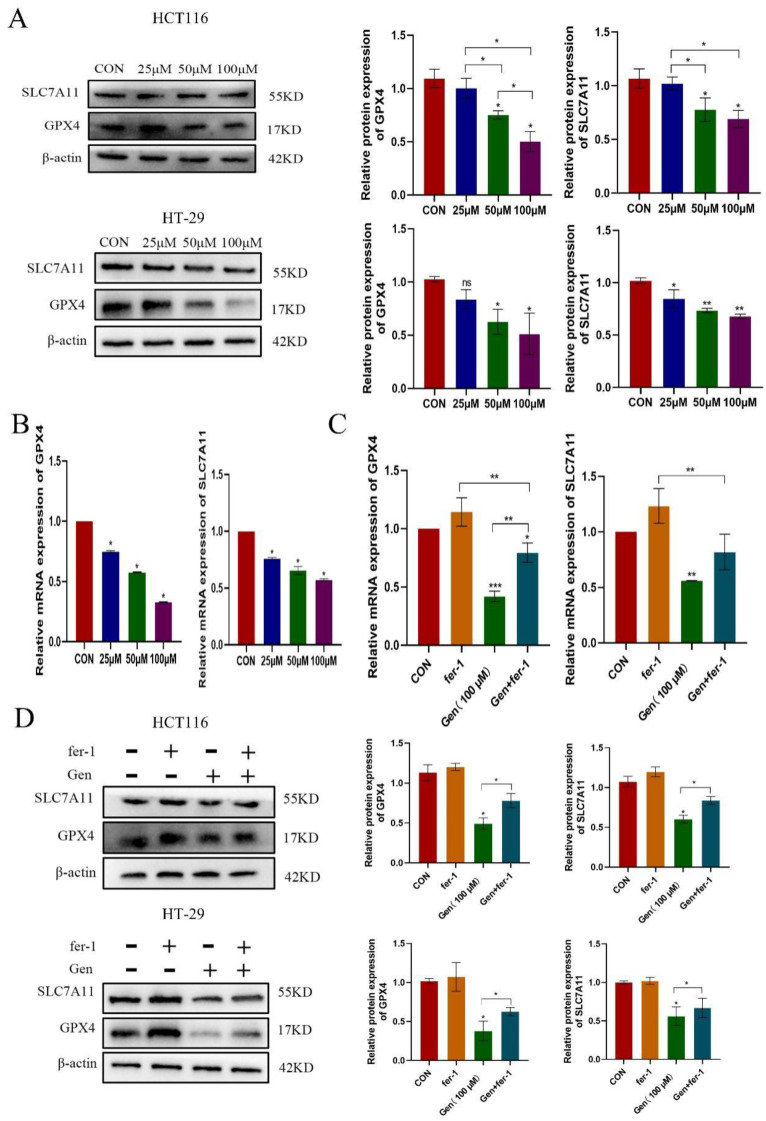
** Gen promotes ferroptosis in CRC cells by affecting SLC7A11/GPX4. (A)** Changes in the protein levels of SLC7A11 and GPX4 after treatment of HCT116 and HT-29 cells with different concentrations of drugs. **(B-C)** Changes in mRNA levels of SLC7A11 and GPX4 in HCT116 cells in drug concentration gradient and fer-1 co-treatment groups. **(D)** Changes in the protein levels of intracellular SLC7A11 and GPX4 after pretreatment of HCT116 and HT-29with fer-1 (10 μM), a ferroptosis inhibitor, for 12 h and subsequent addition of Gen (100 μM) for 72 h. Protein levels were determined by Western blotting and normalized to β-actin levels. Relative mRNA levels, determined by RT‒qPCR, were normalized to GAPDH levels. Experiments involving mRNA refer to the results obtained from HCT116 cell experiments. The data are presented as the means ± SDs (n = 3). * p < 0.05; **p<0.01.

To confirm the mechanism by which Gen affects ferroptosis in cells, we treated HCT116 and HT-29 cells with different concentrations of Gen and collected cell lysates and total RNA to examine the changes in the ferroptosis-related proteins SLC7A11 and GPX4. We found that the protein (Figure 2A) and mRNA (Figure 2B) levels of SLC7A11 and GPX4 decreased as the drug concentration increased. Next, we pretreated HCT116 and HT-29 cells with 10 μM fer-1 for 12 h and then treated them with 100 μM Gen for 72 h. Fer-1 significantly counteracted the effects of Gen on the protein levels of SLC7A11 and GPX4 (Figure 2D) as well as the mRNA levels (Figure 2C) in HCT116 and HT-29 cells. These results demonstrated that Gen promotes ferroptosis in CRC cells by affecting SLC7A11/GPX4.

### 3.3 Gen promotes ferroptosis in CRC cells by downregulating GPX4

**Figure 3:**
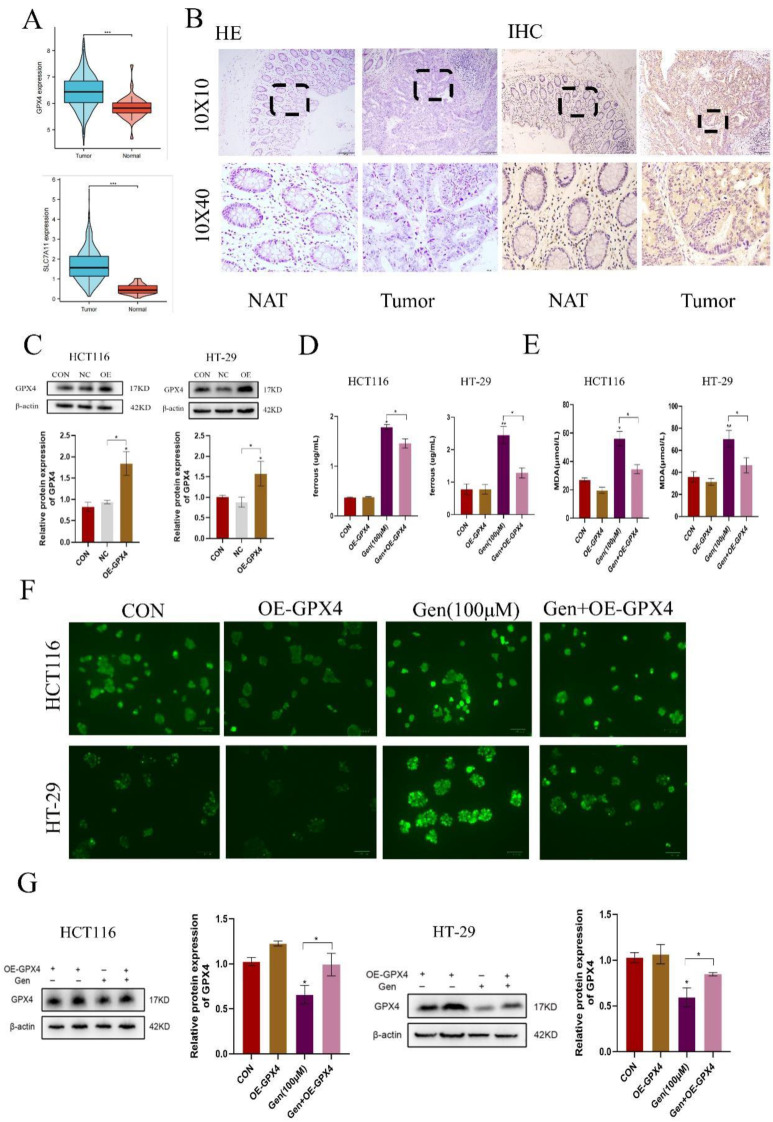
** Gen promotes ferroptosis in CRC cells by downregulating GPX4. (A)** Differential analysis of GPX4 and SLC7A11 in the TCGA database revealed that both are highly expressed in cancer patients. **(B)** GPX4 was also found to be highly expressed in CRC patient samples by immunohistochemistry. **(C)** Overexpression of GPX4 was validated at the protein levels. **(D-F)** Relative levels of intracellular Fe^2+^ **(D)**, MDA **(E)**, and ROS **(F)** after cotreatment with OE-GPX4 and Gen. **(G)** Changes in the relative protein levels of GPX4. Protein levels were determined by Western blotting and normalized to β-actin levels. The data are presented as the means ± SDs (n = 3). * p < 0.05; **p<0.01.

Following drug treatment, we observed that the changes in the expression of GPX4, a classic protein associated with ferroptosis, were more pronounced than those in the SLC7A11 cells at both the mRNA and protein levels. Therefore, we first analyzed the differential expression of GPX4 and SLC7A11 using the TAGG database and found that both were highly expressed in cancer patients (Figure 3A). We also confirmed these findings by analyzing GPX4 in human CRC specimens (Figure 3B). After validating the overexpression of GPX4 (Figure 3C) and cotreating cells with Gen, we observed that OE-GPX4 significantly antagonized the effects of Gen on Fe^2+^, MDA, and ROS levels (Figure 3D-F) as well as on the levels of GPX4 (Figure 3G). Therefore, Gen induces ferroptosis in cancer cells by downregulating GPX4.

### 3.4 The binding specificity coefficient between Gen and FoxO3 is high, and there is a negative correlation between FoxO3 and GPX4

**Figure 4:**
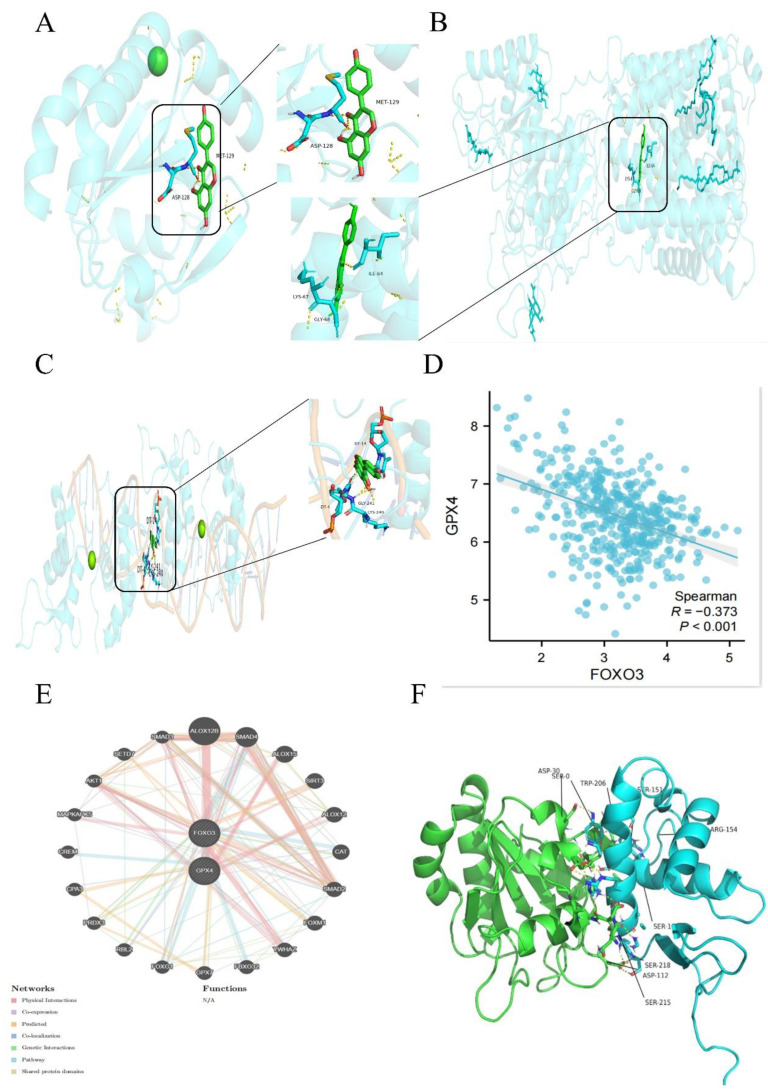
** High Gen and FoxO3 binding specificity coefficients and negative correlation between FoxO3 and GPX4. (A-C)** The specific binding coefficients of GPX4 **(A)**, SLC7A11 **(B)**, and FoxO3 **(C)** were measured using the PubChem database. **(D)** Spearman correlation analysis was performed to explore the coexpression relationships. **(E-F)** The GeneMANIA database was used to identify genes that may interact with GPX4 and FoxO3, and the coexpression network was further evaluated.

To investigate whether Gen directly affects GPX4 to influence ferroptosis, we obtained the structures of the small molecule ligands and target protein receptors from the PubChem and PDB databases. Preprocessing was conducted using PyMOL and AutoDock software, and the active pockets were identified. Molecular docking was employed to predict the binding modes of the small molecule ligands and target macromolecular receptors. The docking results were evaluated based on binding energy, focusing on key target proteins. The specificity coefficient of the drug binding to GPX4 and SLC7A11 was not high (Figure 4A-B), while the specificity coefficient with the transcription factor FoxO3 was as high as 9.5 (Figure 4C). Detailed tabular data can be found in the Supplementary Materials (S1-S3). The COAD dataset retrieved from the TCGA database was analyzed, and the expression data of "GPX4" and "FoxO3" in tumor patients were extracted. Spearman correlation analysis was used to explore the coexpression relationships of the genes. These parameters were negatively correlated (Figure 4D). Additionally, the GeneMANIA database was used to identify genes that may interact with GPX4 and FoxO3, and the coexpression network was further evaluated, suggesting the possible existence of other intermediate mediators (Figure 4E and 4F).

### 3.5 Gen promotes ferroptosis by activating FoxO3

**Figure 5:**
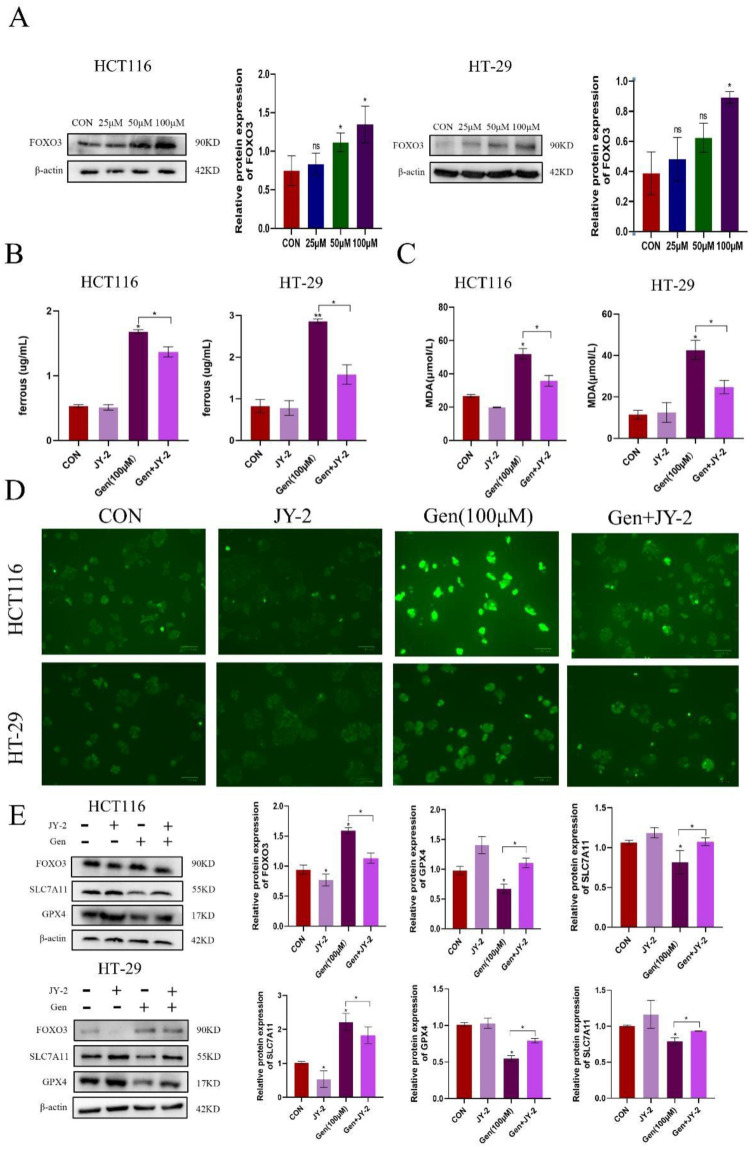
** Gen promotes ferroptosis by activating FoxO3. (A)** Changes in the expression of FoxO3 after treatment of HCT116 and HT-29 cells with different concentrations of Gen. **(B-D)** Relative levels of intracellular Fe^2+^ **(B)**, MDA **(C)**, and ROS **(D)** after cotreatment of HCT116 and HT-29 cells with the FoxO3 inhibitor JY-2 and Gen. **(E)** Relative levels of protein of FoxO3, GPX4, and SLC7A11 in cells after cotreatment of HCT116 and HT-29 cells with the FoxO3 inhibitor JY-2 and Gen. The protein concentrations were determined by Western blotting and normalized to the β-actin level. The data are presented as the means ± SDs (n = 3). * p < 0.05; **p<0.01.

After treating HCT116 and HT-29 cells with different concentrations of Gen, the expression level of the transcription factor FoxO3 gradually increased (Figure 5A). Subsequently, we cotreated HCT116 and HT-29 cells with the FoxO3 inhibitors JY-2 and Gen and found that JY-2 antagonized the impact of Gen on ferroptosis (Figure 5B-D), as did the mRNA and protein levels of SLC7A11 and GPX4 in HCT116 and HT-29 cells (Figure 5E). In summary, Gen promotes ferroptosis in CRC cells through the FoxO3/SLC7A11/GPX4 signaling pathway, achieving an antitumor effect.

## 4. Discussion

Iron-dependent cell death, known as ferroptosis, is influenced by iron metabolism, lipid metabolism, and amino acid metabolism. The XC-/GPX4 pathway is the most extensively studied regulatory system 30. The XC- system primarily consists of solute carrier family 3 member 2 (SLC3A2) and solute carrier family 7 member 11 (SLC7A11, also known as xCT). SLC7A11 plays a key role in transporting cystine into cells, which is subsequently utilized for the synthesis of glutathione. Glutathione is a crucial antioxidant that counteracts the accumulation of reactive oxygen species during ferroptosis. GPX4, utilizes glutathione as a reducing cofactor to detoxify lethal reactive oxygen species and protects cells from oxidative damage 31. Due to the promising application of ferroptosis in cancer therapy and overcoming drug resistance, ferroptosis has become a potential target for various types of therapeutic agents. Gen exhibits antitumor activity in CRC cells and shares common characteristics with ferroptosis, such as lipid peroxidation accumulation and an impact on antioxidant enzymes upon its introduction. Therefore, in this study, we investigated whether ferroptosis plays a role in the antitumor activity of Gen and how it exerts its effects. Our research results demonstrated that Gen induces ferroptosis by inhibiting the FoxO3/SLC7A11/GPX4 pathway, thus inhibiting the proliferation of CRC cells.

Gen can play a therapeutic role in the prevention and treatment of a variety of diseases. Some studies have shown that the risk of CRC in Asian populations is significantly lower than that in Western countries, possibly due to the characteristics of the Asian diet. Asians consume a wide range of soybean products, and the main ingredient in soybeans is Gen (phytoestrogens), which not only plays a preventive role in CRC but also against diabetes, cardiovascular function, adenomatous polyposis, and other diseases 32. A large body of literature has also proven the therapeutic effects of Gen, and our findings are consistent with those of our predecessors in terms of its antiproliferative effects on CRC tumors. In addition, our study revealed that the levels of MDA, Fe^2+^, and ROS, which are related indicators of ferroptosis, increased with increasing concentration, and the levels of GPX4 and SLC7A11, which are related proteins of the XC^-^/GPX4 pathway, decreased after GEN was applied to CRC cells. Moreover, when Gen was used in combination with fer-1, a ferroptosis inhibitor, the viability of HCT116 and HT-29 cells increased, and the levels of MDA, Fe^2+^, and ROS decreased. Therefore, Gen was able to exert anti-CRC effects by inducing ferroptosis.

Therefore, based on our findings, when Gen acts on HCT116 and HT-29 cells, it leads to ROS accumulation, and the oxidative state becomes more apparent at higher concentrations. However, in a study by Vasudevan Sekar *et al.* 17, Gen activated the Nrf-2/HO-1 signaling pathway to reduce cellular oxidative stress, thereby inhibiting carcinogenesis. This difference may be caused by the fact that the mice were treated with a drug that was administered by gavage. The gastrointestinal barrier can affect the absorption of drug concentration. Due to the low bioavailability of Gen, increasing its concentration in the serum can improve its anticancer effects. There have been reports of the development of an immune conjugate (Gen-17. A) composed of Gen and monoclonal antibody 17. A, which can maintain the Gen concentration to a certain extent and enhance its antitumor activity 33. Gen-PEG-SiHNM (Gen-loaded polyethylene glycol-modified silica hybrid nanomaterial) enhances the antioxidant and antiproliferative effects of Gen on HT29 human colon cancer cells by regulating endogenous antioxidant enzymes and H2O2 production. Furthermore, the synthesized Gen-PEG-SiHNM also reduces the incidence of side effects 34. Therefore, the antioxidative or pro-oxidative effects of Gen may be related to the concentration of the drug administered. In this study, the drug was directly applied to the cancer cells, achieving an effective concentration that directly acts on the cancer cells.

Figure 1G-H shows that the decrease in GPX4 expression was significantly greater than that in SLC7A11 expression at both the mRNA and protein levels. Therefore, we believe that Gen not only affects the antioxidant function of GPX4 by affecting solely cysteine transport through SLC7A11 but also affects the expression of GPX4 through complex mechanisms. However, Gen-induced ferroptosis in CRC cells is clearly related to GPX4 and SLC7A11. Therefore, we validated their relationship through the overexpression of GPX4 (OE-GPX4) in the opposite direction. After OE-GPX4, a trend similar to that of fer-1 was observed, indicating that Gen can induce ferroptosis by downregulating GPX4.

Ferroptosis is accompanied by the production of a large amount of ROS, which is closely related to mitochondrial dysfunction 35. When mitochondrial function is impaired, ATP production decreases, which activates the AMPK/FoxO3 pathway to participate in the regulation of mitochondrial function and energy homeostasis. Forkhead box O3 (FoxO3) is a transcription factor involved in the regulation of various physiological processes, such as cell metabolism, mitochondrial function, and oxidative stress 36. To explore whether Gen directly affects GPX4 to induce ferroptosis, we used molecular docking tools and found that the drug had a low specific binding coefficient with GPX4 and SLC7A11, while the coefficient for the transcription factor FoxO3 was as high as 9.5. It was also found that FoxO3 is negatively correlated with GPX4 and that there are other intermediate mediators affecting them. In addition, a study on energy stress showed that FoxO3 can directly bind to the promoter of SLC7A11 to inhibit its expression and regulate glutamate metabolism 37. However, whether FoxO3 in CRC affects changes in SLC7A11 and GPX4 to regulate ferroptosis is still unclear. Therefore, we detected changes in the content of FoxO3 in CRC cells treated with different concentrations of Gen and found that the expression of FoxO3 increased with increasing concentrations of the drug. Furthermore, after treatment with JY-2, a FoxO3 inhibitor, the expression of GPX4 and SLC7A11, which decreased in response to Gen treatment, increased, and the expression of ROS, MDA, and Fe^2+^ also decreased, indicating that Gen promotes ferroptosis by inhibiting the expression of SLC7A11 and GPX4 through activation of FoxO3.

Therefore, the antitumor effect of Gen is significant. However, this drug is currently rarely used in clinical treatment and remains in the development stage. In addition to using it alone, it may also be considered in combination with first-line drugs for colorectal cancer treatment, such as oxaliplatin and 5-FU, to enhance therapeutic efficacy. As Gen is a natural compound with high drug safety, combining it with other drugs might increase its efficacy and reduce the side effects of chemotherapy drugs, which will be a key focus of future research. Additionally, during further *in vivo* and clinical trials, appropriate modifications to the administration route should be made to avoid affecting the experimental results due to bioavailability issues.

## 5. Conclusion

In conclusion, our research findings indicate that Gen promotes ferroptosis in CRC cells by activating FoxO3 and further inhibiting the XC^-^/GPX4 axis. Gen, as a naturally occurring compound, not only has disease prevention properties but also shows potential as a CRC therapeutic drug. It offers several advantages, including easy accessibility, economic benefits, broad functionality, and high safety (Gen is classified by the FDA as a substance generally recognized as safe 32), making it a valuable candidate for further development.

## Supplementary Material

Supplementary tables.

## Figures and Tables

**Figure 6:**
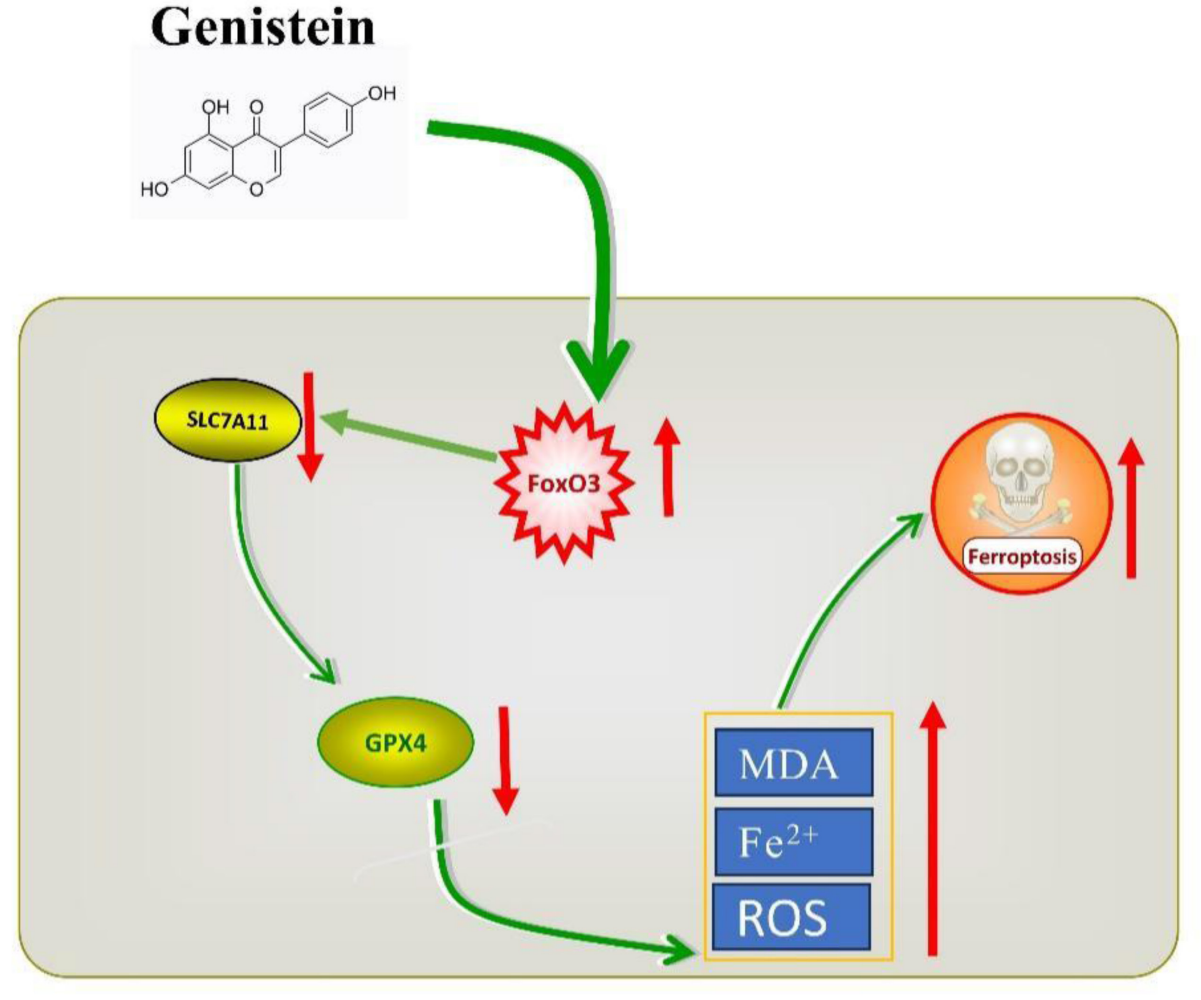
Graphical summary of the current research.

## Abbreviations

Gen: Genistein
ROS: Reactive oxygen species
MDA: Malondialdehyde
Fe^2+^: Ferrous ion
fer-1: ferrostatin-1
RT‒qPCR: Real-time RT-PCR
IHC: Immunohistochemistry
HE: Hematoxylin-Eosin Staining
RIPA: Radio Immunoprecipitation Assay
PVDF: Poly (vinylidene fluoride)
