# Supplementary material for: Genistein Induces Ferroptosis in Colorectal Cancer Cells via FoxO3/SLC7A11/GPX4 Signaling Pathway
Source: J Cancer. 2024 Nov 4;15(20):6741–53. doi: 10.7150/jca.95775 (PMC11632993; doi:10.7150/jca.95775)
Supplement: Supplementary file 1 — Supplementary tables. [file jcav15p6741s1.pdf]

S1. Specific binding coefficient of GPX4 to genistein

mode	affinity (kcal/mol)	dist from best mode rmsd l.b.	dist from best mode rmsd u.b.
1	-7.0	0.000	0.000
2	-6.9	19.339	22.907
3	-6.7	20.905	23.690
4	-6.4	1.620	2.076
5	-6.3	1.716	6.619
6	-6.2	1.763	2.105
7	-6.1	28.614	30.585
8	-6.1	28.372	30.790
9	-6.1	19.344	23.252
10	-5.9	27.816	28.849
11	-5.9	14.122	17.113
12	-5.9	28.411	29.458
13	-5.9	1.301	6.861
14	-5.8	28.188	29.240
15	-5.7	1.844	6.714
16	-5.7	17.051	17.717
17	-5.7	18.953	21.176
18	-5.6	21.952	24.515
19	-5.5	3.908	8.666
20	-5.4	15.951	17.802

Writing output ... done.

S2. Specific binding coefficient of SLC7A11 to genistein

mode	affinity (kcal/mol)	dist from best mode rmsd l.b.	rmsd u.b.
1	-8.0	0.000	0.000
2	-7.9	3.493	5.632
3	-7.8	12.559	13.289
4	-7.8	2.986	5.115
5	-7.7	12.732	14.485
6	-7.7	3.647	6.724
7	-7.7	13.138	14.613
8	-7.6	1.438	6.902
9	-7.1	12.393	14.461
10	-7.1	12.261	14.222
11	-7.0	12.407	13.294
12	-7.0	4.237	6.412
13	-7.0	11.750	12.261
14	-6.9	3.667	6.368
15	-6.9	2.773	3.862
16	-6.9	12.635	13.626
17	-6.9	2.895	6.635
18	-6.8	2.840	6.091
19	-6.7	3.288	6.499
20	-6.6	3.194	4.902

Writing output ... done.

S3. Specific binding coefficient of FoxO3 to genistein

mode	affinity (kcal/mol)	dist from best mode rmsd l.b.	rmsd u.b.
1	-9.5	0.000	0.000
2	-8.9	17.283	21.392
3	-8.5	14.793	17.096
4	-7.8	12.957	17.718
5	-7.7	15.609	17.974
6	-7.7	25.649	27.445
7	-7.6	14.810	17.788
8	-7.6	15.917	19.028
9	-7.5	14.753	17.654
10	-7.5	14.505	19.008
11	-7.4	13.066	16.135
12	-7.3	15.266	16.137
13	-7.3	15.041	15.850
14	-7.3	14.862	17.158
15	-7.2	12.355	14.356
16	-7.2	14.558	17.283
17	-7.2	24.973	27.706
18	-7.2	17.065	19.341
19	-7.1	12.826	15.372
20	-7.1	26.619	28.903

Writing output ... done.
